# Analysis of salt resistance conferred by salt overly sensitive 3 protein from mulberry (*Morus notabilis*)

**DOI:** 10.3389/fpls.2026.1694392

**Published:** 2026-01-30

**Authors:** Dan Liu, Changyu Qiu, Sheng Huang, Xiaomei Lu, Yanrong Zeng, Guangshu Zhu, Chaohua Zhang, Qiang Lin

**Affiliations:** 1 Department of Mulberry Genetic Breeding, Sericulture Technology Promotion Station of Guangxi Zhuang Autonomous Region, Nanning, China; 2 Guangxi Key Laboratory of Sericultural Genetic Improvement and Efficient Breeding, Nanning, China

**Keywords:** growth curves, ion transport, plant genetic engineering, prokaryotic expression, salt overly sensitive, salt stress

## Abstract

**Introduction:**

Soil salinization severely threatens crop productivity and agricultural sustainability. Mulberry (*Morus notabilis*) is an economically important woody crop, but the molecular mechanisms of its salt tolerance remain unclear. The conserved Salt Overly Sensitive 3 (*SOS3*) gene regulates ion homeostasis under salt stress, making it a key target for exploring mulberry’s salt adaptation.

**Methods:**

We cloned *MnSOS3* from *M. notabilis*, constructed a prokaryotic expression vector (pCold-TF-*MnSOS3*) for transformation into *Escherichia coli* BL21, and generated *MnSOS3*-overexpressing transgenic tobacco (*Nicotiana benthamiana*) via *Agrobacterium*-mediated transformation. Salt tolerance was evaluated in *E. coli* (0.6 M NaCl) and tobacco (300 mM NaCl), with Na⁺/K⁺ content quantification in tobacco leaves (250 mM NaCl, 24 h) and verification of MnSOS3-MnSOS2 interaction via yeast two-hybrid assay.

**Results and discussion:**

Recombinant *E. coli* expressing *MnSOS3* showed enhanced growth (maximum OD₆₀₀ = 0.338) vs. near-zero growth in the control. Transgenic tobacco line *MnSOS3*-2 (highest expression) exhibited 33.3% survival under 300 mM NaCl, 2.5-fold higher than wild-type (WT, 13.3%). Transgenic leaves accumulated 54.8% less Na⁺ (0.95 ± 0.02 vs. 2.10 ± 0.07 mmol/g FW) and 45.8% more K⁺ (1.75 ± 0.02 vs. 1.20 ± 0.02 mmol/g FW) than WT, leading to a 67.8% lower Na⁺/K⁺ ratio (0.55 ± 0.01 vs. 1.71 ± 0.08). The yeast two-hybrid assay confirmed MnSOS3-MnSOS2 interaction. These findings show MnSOS3 boosts salt tolerance via Na⁺/K⁺ homeostasis and SOS pathway activation, a key resource for salt-tolerant mulberry breeding.

## 1 Introduction

Salt stress significantly inhibits plant growth and development. When soil salinity exceeds 0.25%, plants suffer from ion imbalance, osmotic stress, and oxidative damage, which can lead to mortality (Silva et al., 2021). To cope with this adversity, plants have evolved a suite of adaptive mechanisms, including osmotic adjustment via accumulating compatible solutes, ion compartmentalization in vacuoles, and reactive oxygen species (ROS) scavenging through antioxidant enzyme systems (Ashraf et al., 2010; Tuteja, 2007). Among these strategies, the perception and transduction of salt stress signals are critical for initiating timely and effective tolerance responses, as they connect external stress stimuli to internal physiological and molecular changes (Yang et al., 2023, 1997).

The Salt Overly Sensitive (SOS) signaling pathway is one of the most well-characterized salt tolerance mechanisms, first identified and extensively studied in the model plant *Arabidopsis thaliana*. This pathway comprises five core genes—*AtSOS1* to *AtSOS5*—that work in a coordinated manner to maintain ion homeostasis (Shi et al., 2002, 2003; Zhu et al., 2010). The pathway is triggered by salt-induced increases in cytosolic Ca²^+^ concentration, which are sensed by SOS3, a calcineurin B-like (CBL) protein. Upon binding Ca²^+^, SOS3 undergoes a conformational change and interacts with SOS2, a serine/threonine protein kinase, forming an active SOS3–SOS2 kinase complex (Qiu et al., 2002). This complex then phosphorylates and activates SOS1, a plasma membrane-localized Na^+^/H^+^ antiporter that mediates the extrusion of excess Na^+^ from the cell, thereby reducing cytotoxic Na^+^ accumulation (Qiu et al., 2002). SOS4, a pyridoxal kinase, is also involved in salt tolerance and root hair development (Shi and Zhu, 2002), highlighting the complexity of the SOS pathway. Structurally, *AtSOS3* contains three conserved EF-hand domains (critical for Ca²^+^ binding) and an N-terminal myristoylation motif (MGXXXS/T(K)); this myristoylation modification is essential for SOS3’s membrane localization and subsequent activation of SOS2 (Ishitani et al., 2000).

Homologs of *SOS3* have been identified in a wide range of plant species, spanning both dicots and monocots, and their structural and functional conservation highlights the evolutionary importance of the SOS pathway. The SOS pathway is conserved across species, such as maize (Wang et al., 2007) and tall fescue (Ma et al., 2014), where SOS gene overexpression enhances salt tolerance. For instance, in rice (*Oryza sativa*), *OsSOS3* enhances salt tolerance by regulating Na^+^ exclusion and maintaining K^+^ homeostasis (Martínez-Atienza et al., 2007); in grapevine (*Vitis vinifera*), *VvSOS3* is upregulated under salt stress and contributes to ion balance (Ma et al., 2019); and in barley (*Hordeum vulgare*), the *SOS3* homolog *Nax4* controls an environmentally sensitive Na^+^ exclusion trait (Rivandi et al., 2010). Notably, in the woody halophyte Tamarix hispida (salt cedar), overexpression of *ThSOS3*—a *SOS3* homolog—improves salt tolerance in both transgenic tamarisk and Arabidopsis by dual mechanisms: boosting the activity of antioxidant enzymes (superoxide dismutase, SOD; peroxidase, POD) to scavenge ROS, and reducing cell membrane lipid peroxidation (Liu et al., 2021b). Recent studies have further expanded our understanding of SOS pathway regulation: in Arabidopsis, the SOS pathway core component SOS2 interacts with FREE1 (a key ESCRT-I component) to regulate vacuole fragmentation, enhancing Na^+^ sequestration efficiency under salt stress (Liu et al., 2025); meanwhile, CIPK24 (a SOS2 regulator) mediates salt-induced growth arrest by phosphorylating RAPTOR1B and inhibiting TORC activity, revealing a balance between stress response and plant growth (Osborne, 2025). Additionally, novel regulators such as PLATZ2 (a transcriptional repressor) and the CBL10-CIPK8 kinase complex have been identified to fine-tune SOS pathway activity at transcriptional and post-translational levels, respectively (Ali et al., 2023), emphasizing the complexity of SOS-mediated salt tolerance mechanisms across plant species.

Mulberry (*Morus notabilis*) is an economically important woody crop with diverse uses: it is the primary food source for silkworms (supporting the global sericulture industry) and its leaves, fruits, and roots have long been used in traditional medicine and functional food production (He et al., 2017; Yuan et al., 2015). However, soil salinization poses a growing threat to mulberry cultivation, as it reduces seedling survival, growth rate, and leaf quality. Despite its agricultural and medicinal value, research on mulberry’s salt tolerance mechanisms remains limited. Existing studies have focused primarily on physiological responses such as polysaccharide accumulation (He et al., 2017) and antioxidant activity (Yuan et al., 2015), while the molecular basis of its salt tolerance—particularly the role of the SOS signaling pathway—has not been characterized. Given the conservation of *SOS3* across plant species and its proven role in enhancing salt tolerance, we hypothesized that mulberry also possesses a *SOS3* homolog (designated *MnSOS3)* that contributes to its salt stress response.

In this study, we cloned the *MnSOS3* gene from *Morus notabilis* and analyzed its sequence characteristics. We validated its function through prokaryotic expression in *Escherichia coli* and *Agrobacterium*-mediated overexpression in transgenic tobacco (*Nicotiana benthamiana*). Additionally, we investigated the interaction between MnSOS3 and MnSOS2 (a key kinase in the SOS pathway) using a yeast two-hybrid assay and examined MnSOS3-mediated changes in ion content under salt stress. Our objectives were to: (1) confirm the role of *MnSOS3* in salt tolerance; (2) elucidate its molecular mechanism of action, particularly its involvement in the SOS pathway and ion transport; and (3) provide a valuable genetic resource for the breeding of salt-tolerant mulberry varieties.

## 2 Materials and methods

### 2.0 Reagents and materials

Plant material: 2-month-old *Morus notabilis* seedlings were collected from the Sericulture Technology Promotion Station of Guangxi (Nanning, China), grown in a greenhouse (25°C, 16 h light/8 h dark).
Microorganisms: *Escherichia coli* BL21 (DE3), A*grobacterium tumefaciens* GV3101, yeast strains Y2HGold/AH109 (Clontech) were stored at -80°C in 20% glycerol.
Vectors: pCold-TF (Takara, Cat. No. 3360), pMD19-T (Takara, Cat. No. 6013), pBWA(V)HS (BioVector, Cat. No. BV001), pGBKT7/pGADT7 (Clontech) were used for cloning and expression.
Reagents: TRIzol® reagent (Invitrogen), Power SYBR® Green Master Mix (Thermo Scientific), IPTG (Sigma-Aldrich, Cat. No. I6758), X-α-Gal (Sigma-Aldrich, Cat. No. B4252) were purchased from commercial suppliers.
Primers: All primers (**Supplementary Table S1**) were synthesized by Tsingke Biotechnology (Beijing, China).”

### 2.1 Cloning of *MnSOS3* and sequence analysis

The *MnSOS3* coding sequence was retrieved from the Mulberry Genome Database and NCBI (GenBank ID: EXB84220.1). Full-length primers were designed using Premier 5.0 (see **Supplementary Table S1**). Total RNA was extracted from roots and leaves of 2-month-old mulberry seedlings (n=15, 3 biological replicates, 5 seedlings per replicate) grown in a greenhouse (25°C, 16 h light/8 h dark) using TRIzol^®^ reagent (Invitrogen) and reverse-transcribed into cDNA using a commercial kit (Thermo Scientific). The cDNA was amplified by PCR under the following conditions: 95°C for 3 min; 30 cycles of 95°C for 30 s, 58°C for 30 s, and 72°C for 1 min; followed by a final extension at 72°C for 10 min. The PCR product was cloned into the pMD19-T vector (TaKaRa) and sequenced.

The *MnSOS2* coding sequence was cloned from *Morus notabilis* using the same method as MnSOS3: retrieved from the Mulberry Genome Database (GenBank ID: EXB21296.1), amplified by PCR (primers listed in **Supplementary Table S1**), cloned into the pMD19-T vector, and verified by Sanger sequencing.

Sequence alignment of MnSOS3 with AtSOS3 was performed using ClustalX 2.1. A phylogenetic tree was constructed with MEGA 12 software, which incorporated SOS3/CBL homologs from 24 plant species, including eggplant (*Solanum melongena*), Arabidopsis (*Arabidopsis thaliana*), and rice (*Oryza sativa*). The Neighbor-Joining (NJ) method was employed for tree construction, with 2000 Bootstrap replicates to evaluate branch support. For distance calculation, the Poisson model was used, and gaps or missing data were handled by partial deletion with a 95% site coverage threshold.

### 2.2 qRT-PCR analysis under stress conditions

Mulberry seedlings (n=30, 3 biological replicates, 10 seedlings per replicate) were treated with 250 mM NaCl or 10% PEG6000. The 250 mM NaCl concentration was selected as it represents moderate-to-severe salt stress, matching the salinity level of common saline-alkali soils in agricultural settings and aligning with standard concentrations used in functional studies of SOS family genes (Martínez-Atienza et al., 2007; Liu et al., 2021). For drought stress, 10% PEG6000 was chosen because it induces a moderate osmotic potential (-0.49 MPa) that effectively triggers typical drought-responsive physiological and molecular changes in mulberry and tobacco, while avoiding excessive stress that would cause rapid seedling death (Ma et al., 2019; Yang et al., 2023). Leaf samples were collected at 0, 8, 24, 32, and 48 h post-treatment. Total RNA was extracted using TRIzol^®^ reagent (Invitrogen, Cat. No. 15596026) following the manufacturer’s protocol, with DNase I (Thermo Scientific, Cat. No. EN0521) treatment to remove genomic DNA (Liu et al., 2022). qRT-PCR was performed on an Applied Biosystems StepOnePlus™ Real-Time PCR System using Power SYBR^®^ Green Master Mix (Thermo Scientific, Cat. No. 4367659). The program was: 95°C for 10 min; 40 cycles of 95°C for 15 s, 58°C for 30 s, 72°C for 30 s; followed by a melting curve analysis (95°C for 15 s, 60°C for 1 min, 95°C for 15 s) to confirm primer specificity (Liu et al., 2021). The mulberry A3 gene (GenBank ID: EXB84221.1) was used as the internal reference for normalizing gene expression (Liu et al., 2021a).

### 2.3 Prokaryotic expression of MnSOS3

The *MnSOS3* gene was inserted into the pCold-TF vector via homologous recombination. Recombinant plasmid pCold-TF-MnSOS3 production required three preconditions: (1) Plasmid construction: The *MnSOS3* gene was inserted into the pCold-TF vector via homologous recombination using the ClonExpress^®^ II One Step Cloning Kit (Vazyme, Cat. No. C112), with a recombination efficiency of 95%; (2) Competent cell preparation: Escherichia coli (E. coli) DH5α competent cells were prepared by the CaCl_2_ method (OD_600_ = 0.4–0.6, 0.1 M CaCl_2_, 15% glycerol) and stored at -80°C; (3) Transformation and screening: Recombinant plasmids were transformed into DH5α competent cells, plated on LB agar medium supplemented with ampicillin (Amp, 100 μg/mL), and positive clones were verified by PCR and Sanger sequencing (100% positive rate). The recombinant plasmid pCold-TF-MnSOS3 was transformed into *E. coli* BL21 (DE3) via the chemical heat shock method, following these steps: (1) 1 μg of plasmid DNA was mixed with 100 μL of competent *E. coli* BL21 (DE3) cells and incubated on ice for 30 min; (2) Heat shock was performed at 42°C for 45 s, followed by immediate transfer to ice for 2 min (ice incubation); (3) 900 μL of LB medium was added, and the mixture was incubated at 37°C for 1 h with shaking at 200 rpm for bacterial recovery; (4) The culture was centrifuged at 5000 × g for 5 min, the pellet was resuspended in 100 μL of LB medium, plated on LB agar medium supplemented with ampicillin (Amp, 100 μg/mL), and incubated at 37°C for 12-16 h. The resulting recombinant plasmid (pCold-TF-*MnSOS3*) was transformed into *E. coli* BL21 (DE3) cells. Protein expression was induced with 0.5 mM IPTG in the presence of Ca²^+^ at 28°C for 8 h. The recombinant protein was purified as previously described (Liu et al., 2022), and its molecular weight was confirmed by SDS-PAGE. SDS-PAGE was performed using 12% separating gel (30% acrylamide/bis-acrylamide, 1.5 M Tris-HCl pH 8.8, 10% SDS, 10% APS, TEMED) and 5% stacking gel (30% acrylamide/bis-acrylamide, 0.5 M Tris-HCl pH 6.8, 10% SDS, 10% APS, TEMED). Electrophoresis was run at 80 V for 30 min (stacking gel) and 120 V for 90 min (separating gel), then stained with Coomassie Brilliant Blue R-250 (Sigma-Aldrich) for 2 h, destained with 10% acetic acid/40% methanol.

### 2.4 Salt stress assay for recombinant *E. coli*

Recombinant (harboring pCold-TF-*MnSOS3*) and control (harboring empty pCold-TF vector) *E. coli* strains were cultured in LB medium supplemented with 0, 0.2, 0.4, or 0.6 M NaCl. Bacterial growth was monitored by measuring the optical density at 600 nm (OD_600_) every 2 h to plot growth curves. Three independent biological replicates were performed for each treatment.

### 2.5 Generation of transgenic tobacco and salt stress assay

*Nicotiana benthamiana* was selected as the eukaryotic model for three reasons: (1) It is a widely used heterologous expression system for woody plant genes (Liu et al., 2019) with high transformation efficiency (80–90% for leaf disc method), enabling rapid generation of transgenic lines; (2) Compared to Arabidopsis (annual herb), tobacco has a similar leaf structure and ion transport mechanism to mulberry (perennial woody plant), reducing species-specific differences in gene function; (3) Maize (monocot) was excluded due to lower transformation efficiency (30–40%) and longer growth cycle (2–3 months vs. 4–6 weeks for tobacco), which is unfavorable for preliminary functional verification.

The *MnSOS3* coding sequence was cloned into the pBWA(V)HS vector and transformed into *Agrobacterium tumefaciens* strain GV3101. Transgenic tobacco (*Nicotiana benthamiana*) plants were generated via the leaf disc transformation method (Liu et al., 2019). Positive transgenic lines were identified by PCR using *MnSOS3*-specific primers (**Supplementary Table S1**) and confirmed by Sanger sequencing with pBWA (V) HS vector primers (**Supplementary Table S1**).

Transgenic lines (*MnSOS3-1* to *MnSOS3-11*) and wild-type (WT) tobacco plants were treated with Hoagland’s solution containing 100, 200, or 300 mM NaCl. Phenotypic responses were documented, and survival rates were calculated after 7 days of treatment. Leaf samples were collected after 7 days for RNA extraction and qRT-PCR analysis. The experiment consisted of three biological replicates, with 10 plants per replicate.

### 2.6 Determination of ion content

The Na^+^ and K^+^ concentrations in the leaves of transgenic and WT tobacco plants under 200 mM NaCl stress were measured using a flame photometer (Sherwood M410). Leaf samples (0.5 g) were dried at 80°C for 48 h, ground into powder, and digested with 5 mL of concentrated HNO_3_ at 120°C for 3 h. The digest was diluted to 50 mL with deionized water, and Na^+^/K^+^ concentrations were measured using a Sherwood M410 flame photometer (Sherwood Scientific, UK) calibrated with standard solutions (1000 ppm Na^+^/K^+^). The measurement was performed in triplicate for each sample (Martínez-Atienza et al., 2007).

### 2.7 Verification of MnSOS3 and MnSOS2 interaction

#### 2.7.1 Media formulations

Media formulations for *E. coli* and yeast are provided in **Supplementary Table S3**.

#### 2.7.2 Yeast two-hybrid assay

The coding sequences of *MnSOS3* (bait) and *MnSOS2* (prey) were cloned into the pGBKT7 and pGADT7 vectors, respectively. The bait and prey plasmids were co-transformed into the yeast strain Y2HGold (purchased from Clontech Laboratories, Inc. (Mountain View, CA, USA), and stored at -80°C in 20% glycerol). Protein–protein interactions were assessed by growth on selective media (SD/-Leu/-Trp/-His/-Ade; QDO) and activation of the MEL1 reporter gene (blue coloration in the presence of X-α-Gal).

#### 2.7.3 Yeast transformation and interaction verification

A single colony of the AH109 yeast strain was inoculated into 50 mL of 2×YPDA liquid medium and cultured at 30°C with shaking at 225 rpm for 12–16 h. When the OD_600_ reached 0.4–0.8, cells were harvested by centrifugation at 700 × g for 5 min. The pellet was washed with sterile water and resuspended in 2.5 mL of sterile water. A PEG/LiAc master mix was prepared by combining 1.2 mL of 50% PEG3350, 180 μL of 1 M LiAc, and 125 μL of denatured single-stranded carrier DNA (10 mg/mL). For each transformation, 1 μg of plasmid DNA was mixed with 300 μL of the PEG/LiAc master mix and 100 μL of the yeast cell suspension. The mixture was incubated at 42°C for 45 min (vortexed every 15 min). Cells were then pelleted, resuspended in 300 μL of sterile water, and spread onto appropriate SD dropout plates. The plates were incubated at 30°C for 3–5 days. To verify interactions, positive colonies were resuspended in sterile water, spotted onto DDO, TDO/X, and QDO/X plates, and incubated at 30°C for 3–5 days.

### 2.8 Statistical analysis

All data are presented as the mean ± standard deviation (SD) of three biological replicates combined with three technical replicates (all experiments have been re-verified to meet this replicate requirement). Statistical significance was determined by one-way analysis of variance (ANOVA) followed by Tukey’s *post-hoc* multiple comparison test, with a p-value < 0.05 considered statistically significant. Statistical parameters (including replicate design, analytical method, and significance threshold) have been clearly specified in this Section 2.8 and the legends of all corresponding figures and tables.

## 3 Results

### 3.1 Cloning and sequence analysis of MnSOS3

**Figure 1:**
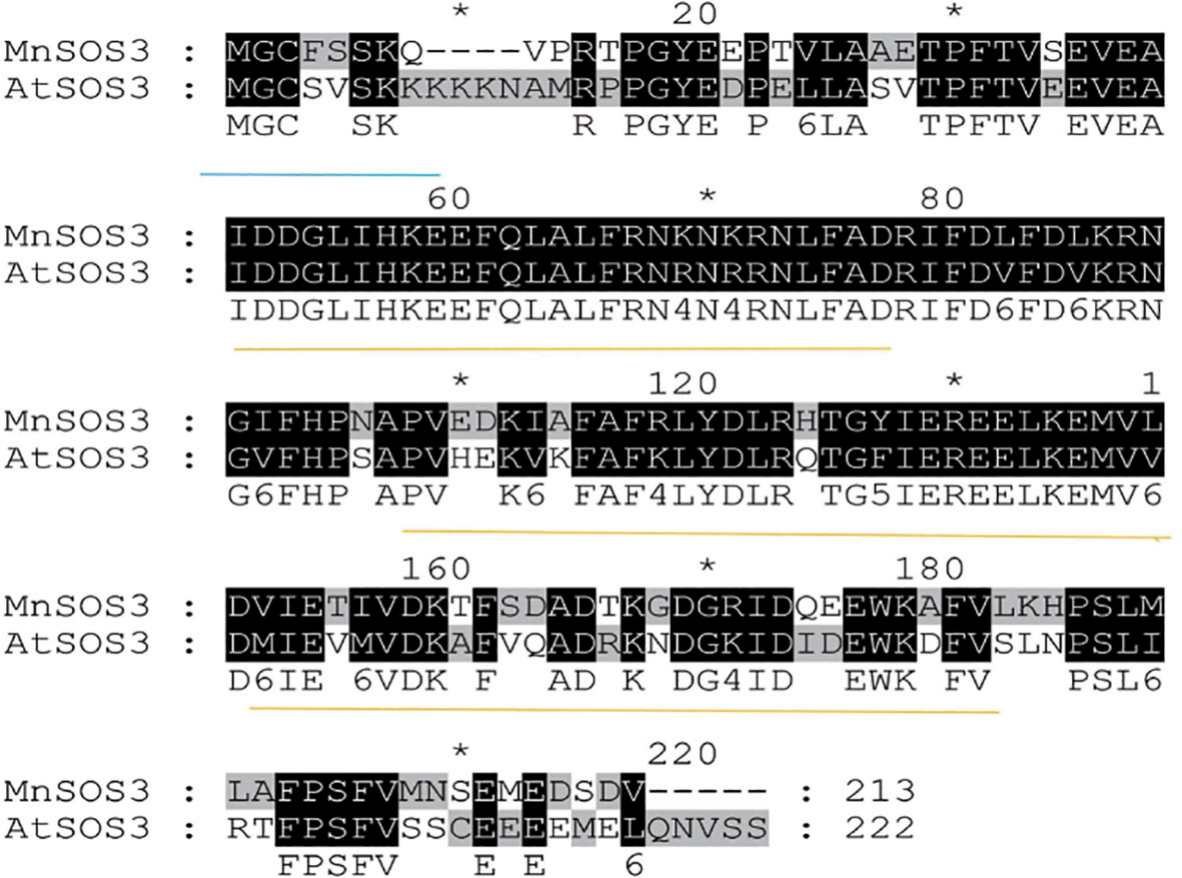
Alignment of MnSOS3 and AtSOS3. Blue underline: myristoylation motif; yellow underline: EF-hand domain.

**Figure 2:**
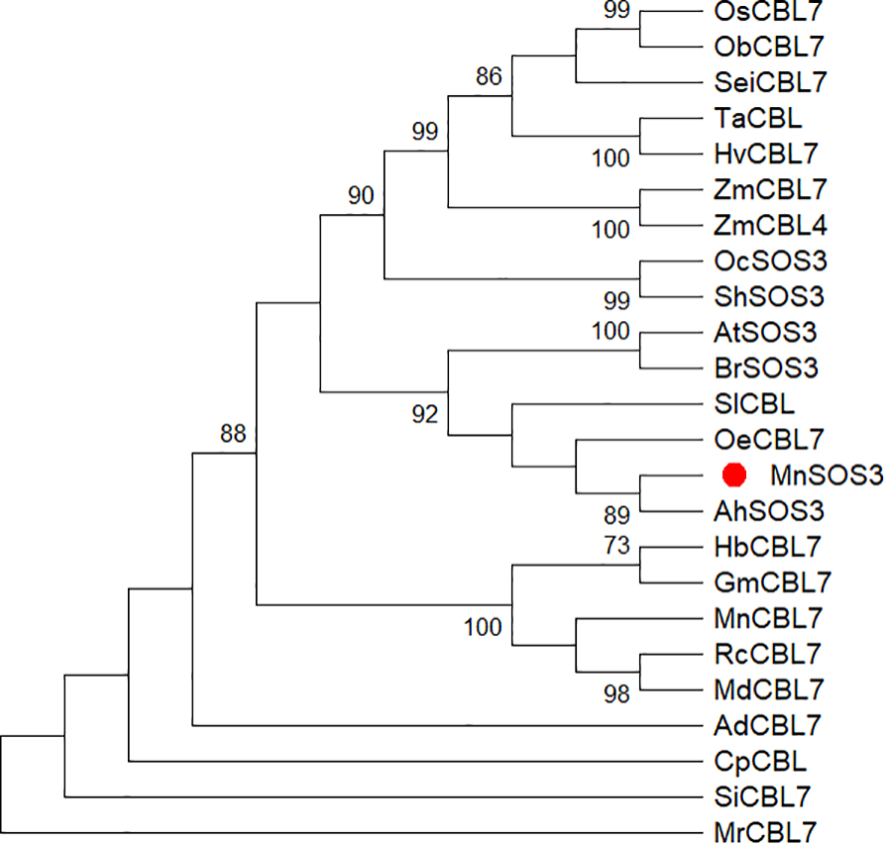
Phylogenetic tree of MnSOS3 and SOS3/CBL homologs from 24 plant species, constructed by Neighbor-Joining method with 2000 bootstrap replicates. The red circle represents the salt overly sensitive 3 of mulberry (MnSOS3). Oc, *Oryza coarctata*; Sh, Saccharum hybrid cultivar; Oc, *Olea europaea* var. *Sylvestris*; Sl, *Solanum lycopersicum*; Mn, *Morus notabilis C.K. Schneid*; Ah, *Arachis hypogaea*; At, *Arabidopsis thaliana*; Br, *Brassica rapa subsp. pekinensis*; Rc, *Rosa chinensis*; Md, *Malus domestic*; Hb, *Hevea brasiliensis*; Gm, *Glycine max*; Cp, *Carica papaya*; Hv, *Hordeum vulgare*; Si, *Sesamum indicum*; Ad, *Arachis duranensis*; Ta, *Triticum aestivum*; Sci, *Setaria italica;* Os, *Oryza sativa Japonica Group*; Ob, *Oryza brachyantha*; Zm, *Zea may*. GenBank number: (OcSOS3, AKA42978.1), (ShSOS3, QJQ27323.1), (OcCBL7, XP_022887032.1), (SlCBL, NP_001234705.1), (MnSOS3, EXB84220.1), (MnCBL7, EXB97803.1), (AhSOS3, XP_025612709.1), (AtSOS3, CCH26627.1), (BrSOS3, AGA95984.1), (RcCBL7,XP_024169307.1), (MdCBL7, XP_028949021.1), (HbCBL7, XP_021686008.1), (GmCBL7, XP_003524435.1), (CpCBL7, XP_021905170.1), (SiCBL7, XP_011082293.1), (AdCBL7, XP_020994235.1), (HvCBL7, KAE8767041.1), (TaCBL, KAF6984752.1), (SciCBL7, XP_022679428.1), (OsCBL7, ABA54182.1), (ObCBL7, XP_015688939.1), (ZmCBL7, PWZ25724.1), (ZmCBL4, NP_001151979.2).

The *MnSOS3* gene contains a 642-bp open reading frame (ORF) encoding a protein of 213 amino acids, with a predicted molecular weight of 24.5 kDa and an isoelectric point (pI) of 4.80 (**Supplementary Figure S1**). The deduced protein sequence contains characteristic EF-hand calcium-binding domains and an N-terminal myristoylation motif (MGCFSSK). MnSOS3 shares 72% amino acid sequence identity with AtSOS3 from *Arabidopsis thaliana* (**Figure 1**). Phylogenetic analysis indicated that MnSOS3 is most closely related to AhSOS3 from peanut (*Arachis hypogaea*) (**Figure 2**).

### 3.2 Expression patterns of *MnSOS3* under abiotic stress

**Figure 3:**
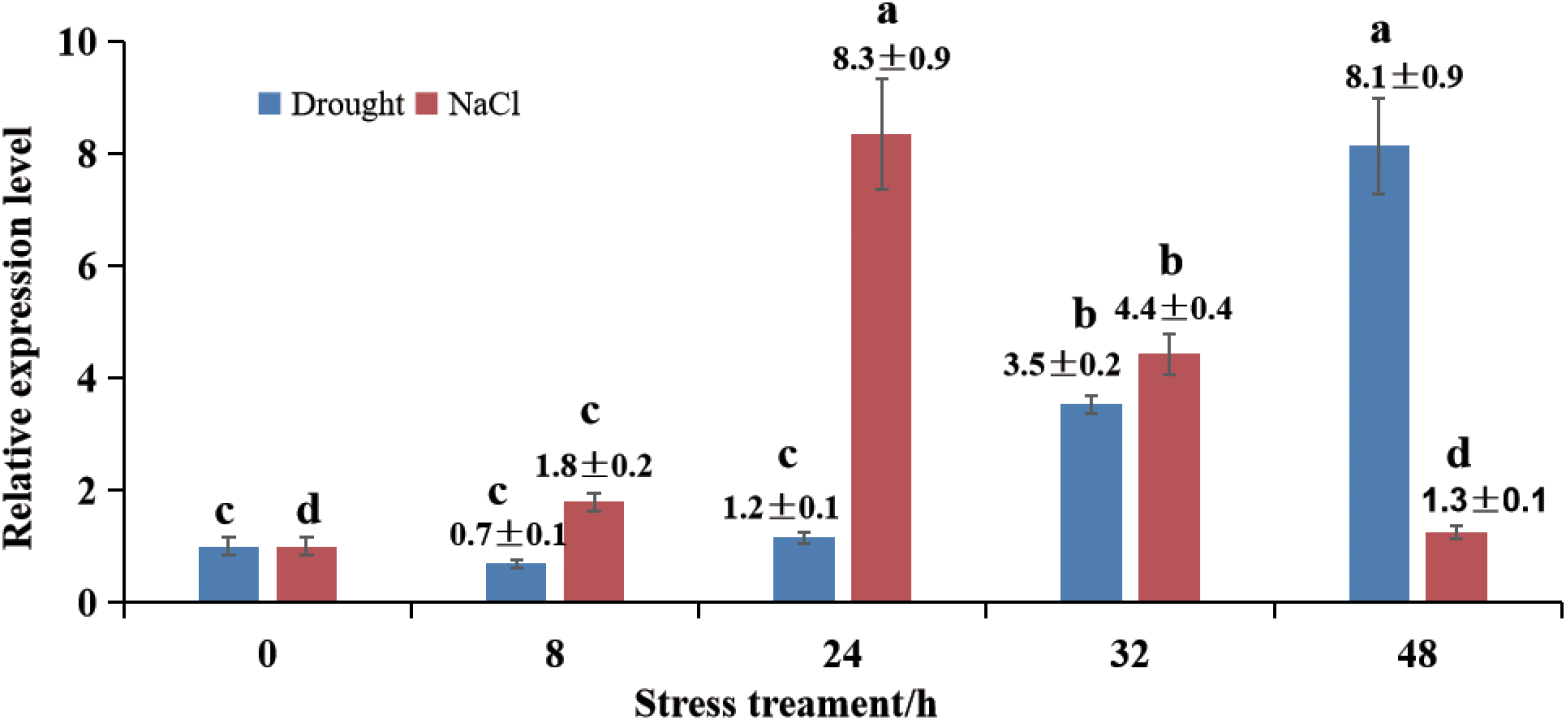
*MnSOS3* expression under stress. **(A)** Salt stress; **(B)** Drought stress. Different lowercase letters above the bars indicate significant differences among groups (p < 0.05, Tukey’s post-hoc test). Data are presented as mean ± SD (n = 3). Control (0 h) expression level was set as 1.

The transcript levels of *MnSOS3* in response to salt and drought stress were analyzed by qRT-PCR. Under salt stress (250 mM NaCl), *MnSOS3* expression was significantly upregulated, peaking at 24 h with a level 8.3-fold higher than that of the untreated control. Expression subsequently declined but remained elevated compared to the control (**Figure 3**). Under drought stress (10% PEG6000), *MnSOS3* expression initially decreased at 8 h but was subsequently induced, reaching a maximum level (8.1-fold higher than the control) at 48 h (**Figure 3**).

### 3.3 Prokaryotic expression and enhanced salt tolerance in *E. coli*

The pCold-TF-*MnSOS3* fusion construct was successfully expressed in *E. coli* BL21 (DE3). SDS-PAGE analysis revealed a single band of approximately 90 kDa for the recombinant protein, consistent with the expected size of the fusion protein (TF tag ~60 kDa + MnSOS3 ~24.5 kDa). The control strain (containing empty vector) expressed only the ~60 kDa TF tag protein (**Supplementary Figure S2**).

**Figure 4:**
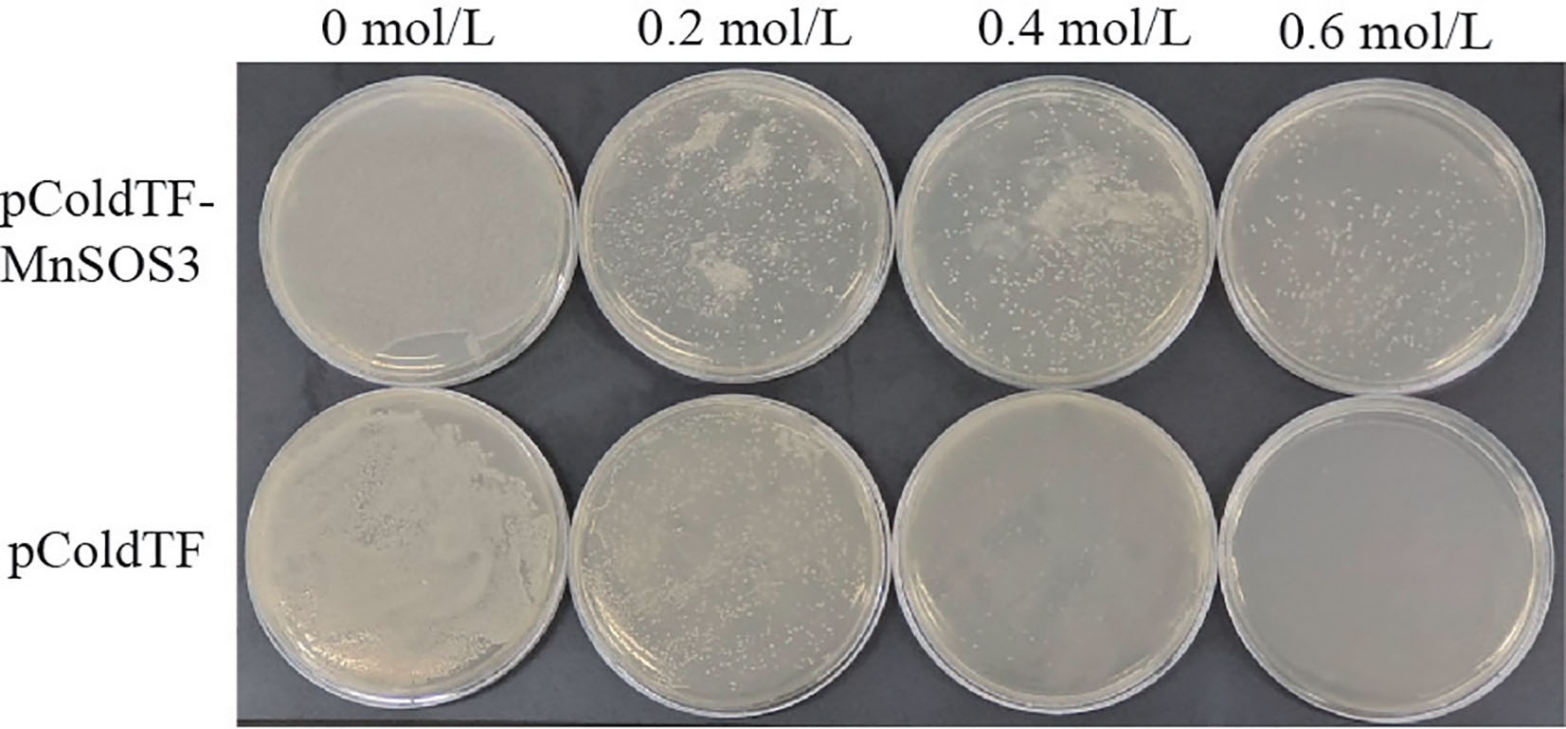
Growth status of *E. coli* BL21 (pCold-TF) and BL21 (pCold-TF-*MnSOS3*) on LB medium containing different concentrations of NaCl.

**Figure 5:**
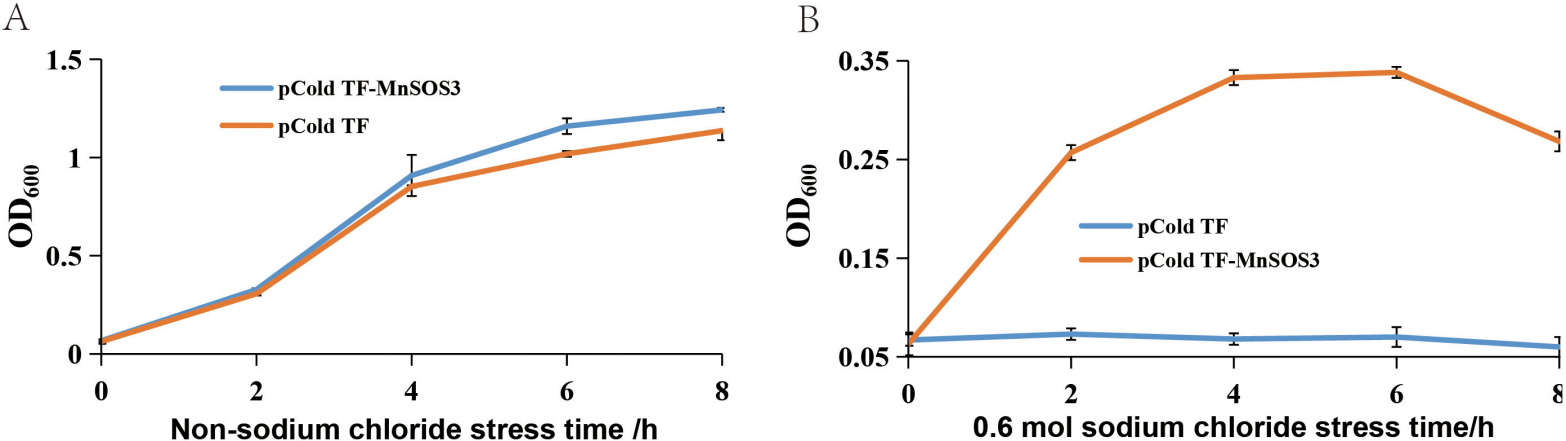
Growth curves of *E*. *coli* BL21 (pCold-TF) and BL21 (pCold-TF-*MnSOS3*) on LB medium. **(A)** 0 mol/L NaCl; **(B)** 0.6 mol/L NaCl.

Both recombinant and control *E. coli* strains grew normally on LB medium containing 0.2 M NaCl. However, as the NaCl concentration increased, the growth of the control strain (pCold-TF) was severely inhibited. Notably, at 0.6 M NaCl, the control strain barely grew, while the recombinant strain (pCold-TF-*MnSOS3*) exhibited significant growth (**Figure 4**). Growth curve analysis in liquid culture showed no significant difference between the two strains under normal conditions (0 M NaCl) (**Figure 5A**). In contrast, under high salt stress (0.6 M NaCl), the growth of the control strain was strongly suppressed, whereas the recombinant strain maintained steady growth, reaching a maximum OD_600_ of 0.338 (**Figure 5B**).

### 3.4 Molecular confirmation of transgenic tobacco plants

**Figure 6:**
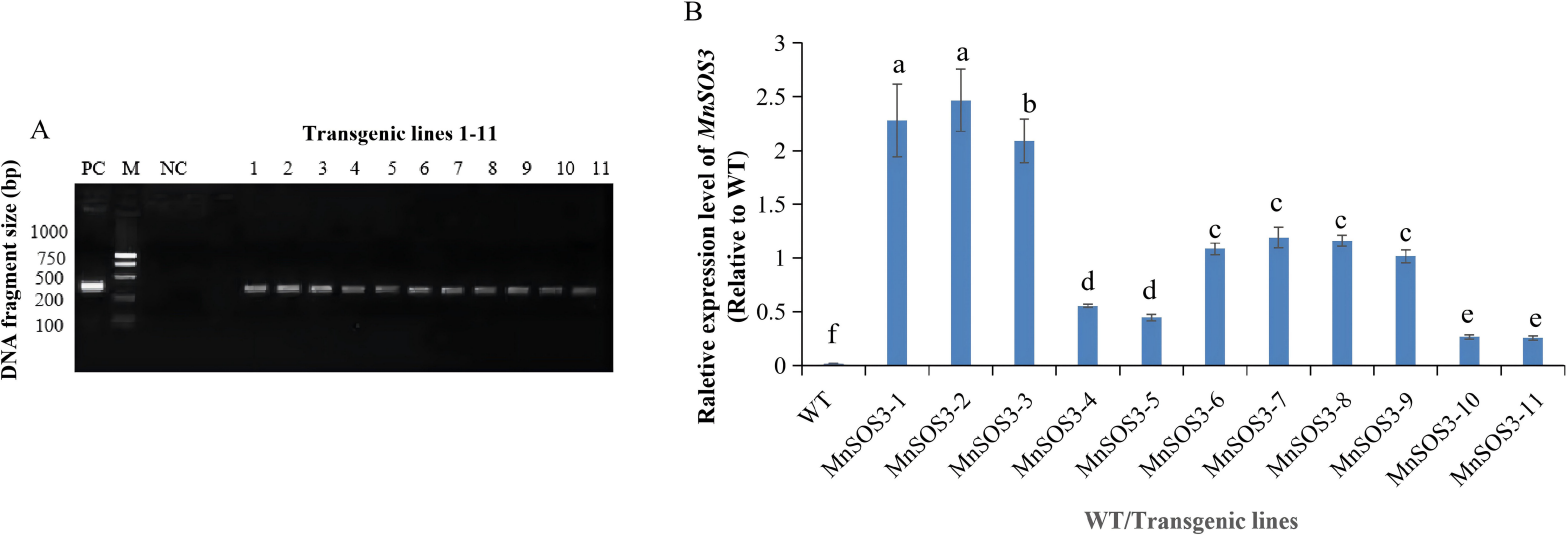
Verification of transgenic tobacco. **(A)** PCR amplification results of MnSOS3 in transgenic tobacco lines. M: DNA Marker (bp); PC: Positive control (recombinant plasmid); NC: Negative control (ddH₂O); 1-11: Transgenic lines *MnSOS3*-1 to *MnSOS3*-11; WT: Wild-type tobacco. **(B)** Relative expression levels of *MnSOS3* in transgenic lines and WT detected by qRT-PCR. Data are presented as mean ± SD (n=3). Different lowercase letters indicate significant differences among groups (P<0.05).

The *MnSOS3*-pBWA(V)HS recombinant plasmid was introduced into tobacco via *Agrobacterium*-mediated transformation (**Supplementary Figure S3**). A total of 11 independent kanamycin-resistant transgenic lines were obtained and designated *MnSOS3-1* to *MnSOS3-11*. PCR amplification using cDNA templates confirmed the integration of the *MnSOS3* transgene in all 11 lines, while no amplification product was detected in wild-type (WT) plants (**Figure 6A**). GUS staining assays confirmed successful transformation, showing clear blue staining in transgenic leaves, while no staining was observed in WT leaves (**Supplementary Figure S4**). qRT-PCR analysis revealed varying expression levels of *MnSOS3* among the transgenic lines, with line *MnSOS3-2* showing the highest expression and *MnSOS3-11* the lowest (**Figure 6B**).

### 3.5 Salt tolerance of transgenic tobacco lines

**Figure 7:**
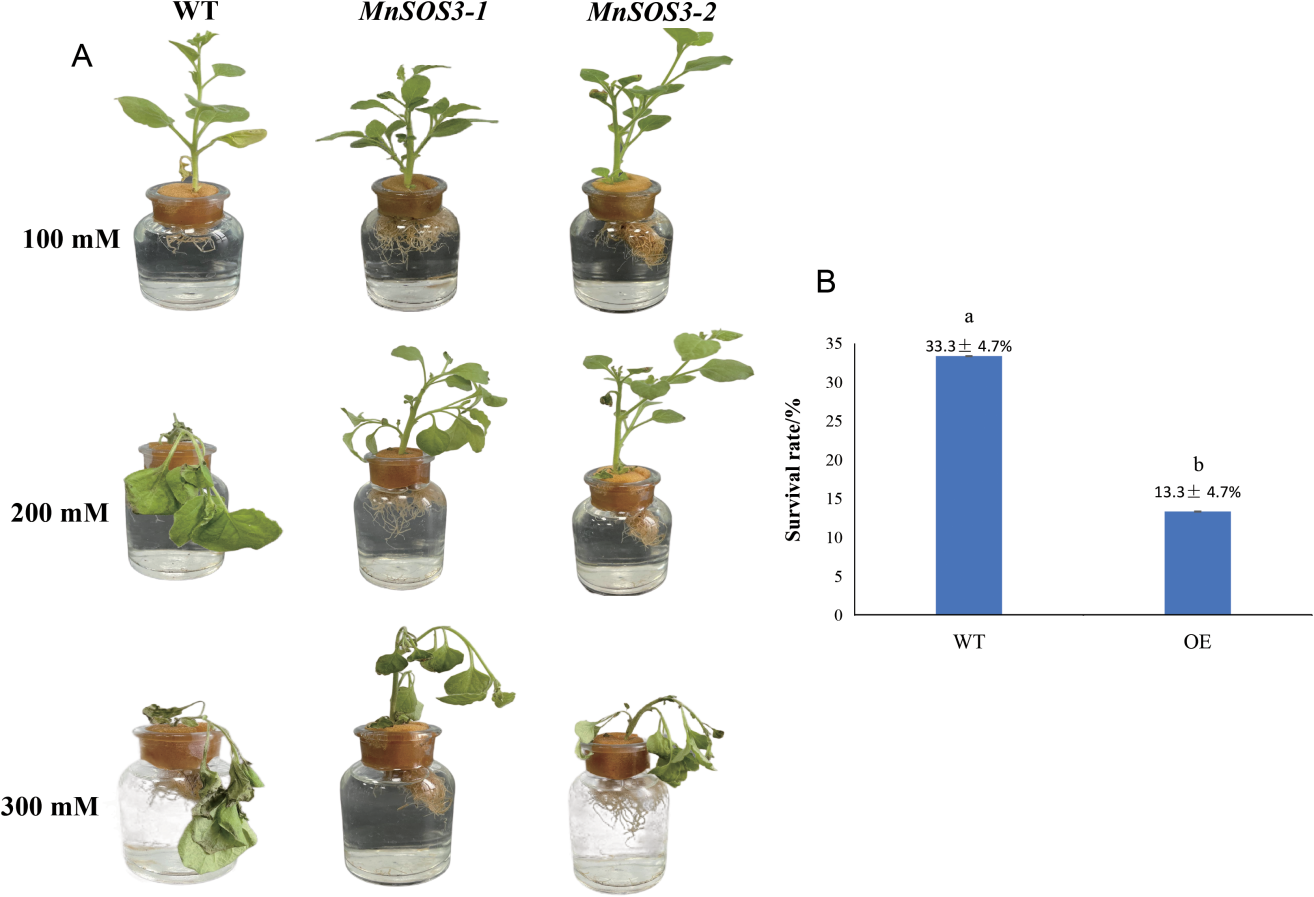
Salt tolerance of transgenic tobacco. **(A)** Phenotypes under 100, 200, 300 mM NaCl for 3 days; **(B)** Survival rates under 300 mM NaCl. Values above bars are mean ± standard deviation (SD, n = 3 biological replicates; 10 plants per replicate, 30 plants in total). Different lowercase letters **(a, b)** indicate significant differences between groups (p < 0.05, Student’s t-test).

Under 100 mM NaCl stress for 5 days, transgenic lines (*MnSOS3*-1 and *MnSOS3*-2) showed no obvious phenotypic abnormalities, whereas wild-type (WT) leaves exhibited slight wilting. Exposure to 200 mM NaCl for 5 days caused minor leaf yellowing in the transgenic lines, while WT leaves displayed severe wilting and chlorosis. Under severe salt stress (300 mM NaCl) for 5 days, all plants showed stress-induced damage, but the transgenic lines maintained viability longer than the WT (**Figure 7A**). After 7 days of 300 mM NaCl treatment, the survival rate of the transgenic lines was 33.3% ± 4.7%, which was significantly higher than that of the WT plants (13.3% ± 4.7%) (**Figure 7B**).

### 3.6 Ion homeostasis in transgenic tobacco under salt stress

The Na^+^ and K^+^ contents were measured in leaves and roots of transgenic (*MnSOS3-2*) and WT plants under 250 mM NaCl stress. Under normal conditions, no significant differences in ion content were observed. After salt treatment, transgenic leaves accumulated significantly less Na^+^ and more K^+^ than WT leaves, resulting in a significantly lower Na^+^/K^+^ ratio. Although transgenic roots accumulated more Na^+^, they maintained a K^+^ level comparable to WT, resulting in a root Na^+^/K^+^ ratio that was not significantly different from that of WT plants (see **Supplementary Table S2**). These results suggest that *MnSOS3* overexpression enhances salt tolerance by modulating ion distribution, potentially sequestering more Na^+^ in the roots to protect photosynthetic tissues in the leaves.

### 3.7 MnSOS3 interacts with MnSOS2 in yeast

**Figure 8:**
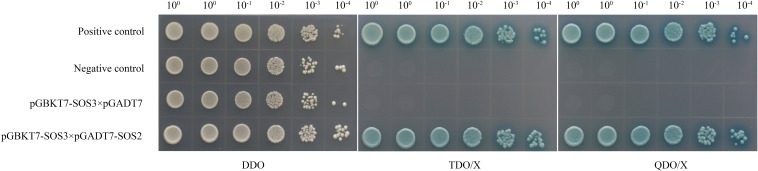
Analysis of SOS3–SOS2 protein interaction by yeast two-hybrid assay on differential dropout media. Double Drop-out (DDO): lacking Tryptophan and Leucine. Triple Drop-out (TDO/X): lacking Trp, Leu, Histidine and supplemented with X-α-gal. Quadruple Drop-out (QDO/X): lacking Trp, Leu, His, Adenine and supplemented with X-α-gal. The growth of blue colonies on TDO/X and QDO/X indicates a positive interaction between SOS3 and SOS2.

A yeast two-hybrid assay was conducted to verify the physical interaction between MnSOS3 and MnSOS2. All control and test groups grew on DDO medium, confirming the successful co-transformation of the bait and prey plasmids. The positive control (pGBKT7-p53 + pGADT7-T) grew and produced blue colonies on the stringent selective media (TDO/X and QDO/X), while the negative control (pGBKT7-Lam + pGADT7-T) and the autoactivation control (pGBKT7-*MnSOS3* + pGADT7) did not, confirming the specificity of the system. Crucially, the yeast cells co-expressing pGBKT7-MnSOS3 and pGADT7-*MnSOS2* grew and turned blue on both TDO/X and QDO/X media, demonstrating a specific interaction between MnSOS3 and MnSOS2 (**Figure 8**).

## 4 Discussion

MnSOS3 exhibits high structural conservation with known SOS3/CBL family proteins, particularly AtSOS3 from *Arabidopsis thaliana*. Similar to ThSOS3 in Tamarix (Liu et al., 2021b) and MnSPDS in mulberry (Liu et al., 2021a), MnSOS3 regulates ion homeostasis via the SOS pathway. The presence of conserved EF-hand motifs and an N-terminal myristoylation site suggests that MnSOS3 retains the fundamental capabilities of calcium sensing and membrane localization, which are essential for initiating the SOS signaling cascade (Ishitani et al., 2000). Phylogenetic analysis positioned MnSOS3 closely to SlCBL from eggplant (*Solanum melongena*), indicating functional conservation within related plant lineages. This conservation is consistent with recent findings that SOS3/CBL proteins across plant species share core functions in SOS pathway activation, while also evolving species-specific regulatory features (Ali et al., 2023). For instance, *Arabidopsis* SOS3 interacts with CIPK24 to modulate TORC-mediated growth balance (Osborne, 2025), and Tamarix *ThSOS3* regulates both ion homeostasis and ROS scavenging (Liu et al., 2021), suggesting that SOS3 homologs may integrate multiple stress response pathways. The unique myristoylation motif (MGCFSSK) in MnSOS3, differing from the canonical sequence (MGXXXS/T(K)) in Arabidopsis, may represent a woody plant-specific adaptation, similar to the functional diversification of SOS3 homologs reported in other woody species (Ali et al., 2023).

**Figure 9:**
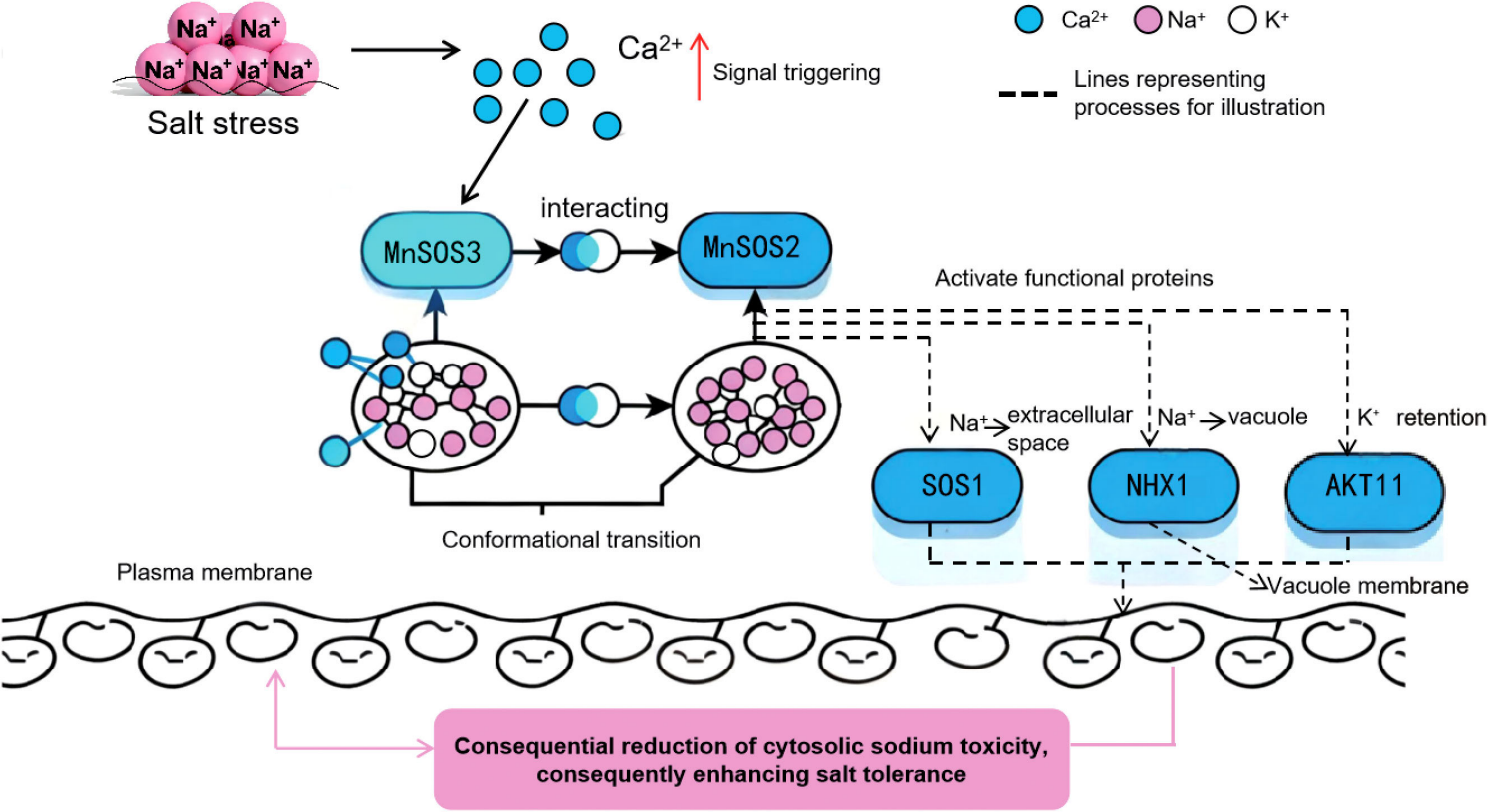
A proposed model of *MnSOS3*-mediated salt tolerance mechanism in mulberry Under salt stress, the increased cytosolic Ca²^+^ concentration (triggered by excessive Na^+^ accumulation) acts as a signal to activate MnSOS3. Activated MnSOS3 undergoes conformational transition and interacts with MnSOS2, forming a functional complex that further activates downstream effector proteins: *SOS1* mediates Na^+^ extrusion from the cytosol to the extracellular space; *NHX1* sequesters cytosolic Na^+^ into vacuoles; *AKT11* maintains intracellular K^+^ homeostasis. Collectively, these processes contribute to the consequential reduction of cytosolic sodium toxicity, consequently enhancing the salt tolerance phenotype of mulberry. Ca²^+^ (blue circle), Na^+^ (pink circle), K^+^ (white circle); dashed lines indicate regulatory processes.

The rapid upregulation of *MnSOS3* under salt stress (**Figure 3**) confirms its role as an early-responsive gene in salt stress signaling. Based on our results and the conserved SOS pathway model, we propose the specific molecular pathway mediated by *MnSOS3* (**Figure 9**):(1) Stress perception and Ca²^+^ signal initiation: Under salt stress, excess Na^+^ influx triggers an increase in cytosolic free Ca²^+^ concentration, a well-characterized secondary messenger in plant salt stress responses (Qiu et al., 2002; Zhu et al., 2010).(2) *MnSOS3* activation via Ca²^+^ binding: The conserved EF-hand domains in MnSOS3 (**Figure 1**) enable specific binding to Ca²^+^, inducing a conformational change in MnSOS3 that exposes its interaction interface with MnSOS2 (Ishitani et al., 2000).(3) Formation of MnSOS3-MnSOS2 kinase complex: Our yeast two-hybrid assay directly confirms the physical interaction between MnSOS3 and MnSOS2 (**Figure 8**). This interaction activates the kinase activity of MnSOS2 by relieving autoinhibition, forming a functional SOS3-SOS2 complex—an indispensable step in SOS pathway activation (Qiu et al., 2002).(4) Modulation of ion transporters for homeostasis: The activated MnSOS3-MnSOS2 complex phosphorylates downstream ion transporters, primarily the plasma membrane-localized Na^+^/H^+^ antiporter SOS1 (Shi et al., 2003). Phosphorylated SOS1 mediates the extrusion of excess Na^+^ from the cytosol to the apoplast, while simultaneously promoting K^+^ retention via regulating K^+^ channels (e.g., AKT1) (Martínez-Atienza et al., 2007). Consistent with this, our ion content analysis shows that transgenic tobacco overexpressing *MnSOS3* accumulates 54.8% less Na^+^ and 45.8% more K^+^ in leaves under salt stress, resulting in a 67.8% lower Na^+^/K^+^ ratio (Abstract; **Supplementary Table S2**). This phenotype directly validates that *MnSOS3* enhances salt tolerance through the canonical SOS pathway by maintaining ion homeostasis. Notably, the root-specific accumulation of Na^+^ in transgenic plants (**Supplementary Table S2**) suggests an additional layer of regulation: *MnSOS3* may also modulate vacuolar Na^+^ sequestration via tonoplast-localized transporters (e.g., *NHX1*) through the SOS3-SOS2 complex, redirecting Na^+^ to roots to protect photosynthetically active leaf tissues—an adaptive strategy reported in woody plants like Tamarix hispida (Liu et al., 2021).This vacuole-mediated Na^+^ sequestration is consistent with recent findings in Arabidopsis, where SOS2 phosphorylates the ESCRT-I component FREE1 to induce vacuole fragmentation, increasing the vacuolar surface-to-volume ratio and enhancing Na^+^ compartmentalization efficiency (Liu et al., 2025). Although we did not directly detect vacuolar dynamics in MnSOS3-overexpressing plants, the observed ion distribution pattern implies that MnSOS3 may regulate vacuolar function through the SOS pathway, similar to the conserved role of SOS2 in mediating endomembrane remodeling under salt stress (Liu et al., 2025). Furthermore, the interaction between MnSOS3 and MnSOS2 aligns with the broader model of SOS pathway activation, where CBL/SOS3 proteins form complexes with CIPK/SOS2 kinases to regulate downstream targets, including ion transporters and vesicular trafficking components (Ali et al., 2023; Osborne, 2025).

The unique myristoylation motif (MGCFSSK) in MnSOS3, differing from the canonical sequence (MGXXXS/T(K)) in *Arabidopsis*, presents an interesting avenue for future research. Site-directed mutagenesis could elucidate how this variation influences membrane targeting, complex formation with SOS2, and overall signaling efficiency. From an application perspective, *MnSOS3* is a promising candidate gene for genetic engineering aimed at improving salt tolerance in mulberry and other economically important crops. Its ability to function heterologously in both bacteria (*E. coli.*) and tobacco indicates a conserved mechanism that could be broadly applicable.

Despite the significant findings, this study has several limitations that should be acknowledged:(1) Heterologous expression system: The functional validation of *MnSOS3* was primarily conducted in transgenic tobacco (*Nicotiana benthamiana*), a heterologous host. While this system effectively demonstrates the gene’s conserved function, the salt tolerance phenotype may differ in homologous mulberry plants due to species-specific genetic backgrounds and regulatory networks. Future studies should generate transgenic mulberry lines to verify *MnSOS3*’s native function.(2) Limited exploration of downstream targets: We confirmed the interaction between MnSOS3 and MnSOS2 but did not identify other potential interacting partners (e.g., additional CBL-interacting protein kinases, CIPKs) or directly validate the phosphorylation of SOS1 by the MnSOS3-MnSOS2 complex. Co-immunoprecipitation (Co-IP) and phosphorylation assays are needed to confirm these downstream signaling events.(3) Single stress type and concentration gradient: This study focused on NaCl-induced salt stress, but natural saline soils often contain mixed salts (e.g., NaHCO_3_, Na_2_SO_4_) that induce both salt and alkaline stress. Additionally, the ion transport mechanism was only analyzed at 250 mM NaCl; exploring multiple salt types and concentration gradients would provide a more comprehensive understanding of MnSOS3’s role.(4) Lack of physiological data on oxidative stress: Previous studies have shown that SOS3 homologs (e.g., ThSOS3) enhance salt tolerance by regulating antioxidant enzyme activity (Liu et al., 2021), but we did not measure ROS levels or antioxidant enzyme (SOD, POD, CAT) activities in transgenic plants. Future work should investigate whether MnSOS3 contributes to oxidative stress tolerance alongside ion homeostasis. Addressing these limitations will deepen our understanding of MnSOS3-mediated salt tolerance and facilitate its application in crop breeding. This study not only deepens our understanding of the salt tolerance mechanism in mulberry but also provides a valuable genetic resource (MnSOS3) for breeding improved salt-tolerant crops.

## 5 Conclusion

In conclusion, we successfully cloned the *MnSOS3* gene from *Morus notabilis* and demonstrated its crucial role in enhancing salt tolerance. *MnSOS3* overexpression improves salinity resilience by modulating the SOS signaling pathway, promoting Na^+^ exclusion, and maintaining K^+^/Na^+^ homeostasis. The interaction between MnSOS3 and MnSOS2 is central to this mechanism. These findings provide valuable insights into the salt tolerance mechanisms of mulberry and establish *MnSOS3* as a prime genetic resource for the breeding of salt-tolerant varieties.

## Data Availability

The original contributions presented in the study are included in the article/**Supplementary Material**. Further inquiries can be directed to the corresponding author.
