# Supplementary material for: Analysis of salt resistance conferred by salt overly sensitive 3 protein from mulberry (Morus notabilis)
Source: Front Plant Sci. 2026 Jan 30;17:1694392. doi: 10.3389/fpls.2026.1694392 (PMC12900689; doi:10.3389/fpls.2026.1694392)
Supplement: Supplementary file 1 [file DataSheet1.docx]

**Supplemental Figures**


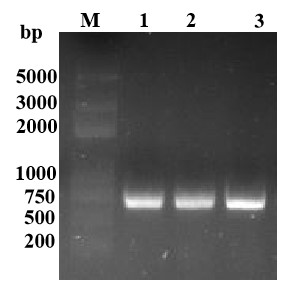


**Supplemental Fig. 1** **PCR amplification of *MnSOS3* gene.**

M: 5000 bp marker 1-3. *MnSOS3.*

**S**
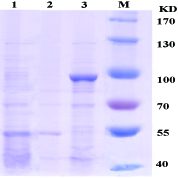


**Supplemental Fig. 2 SDS-PAGE analysis of MnSOS3 protein expressed in *E. coli* BL21 (DE3) cells.** Note: M: 170 kDa protein marker, (a) lanes 1-2: control, lane3 : MnSOS3.


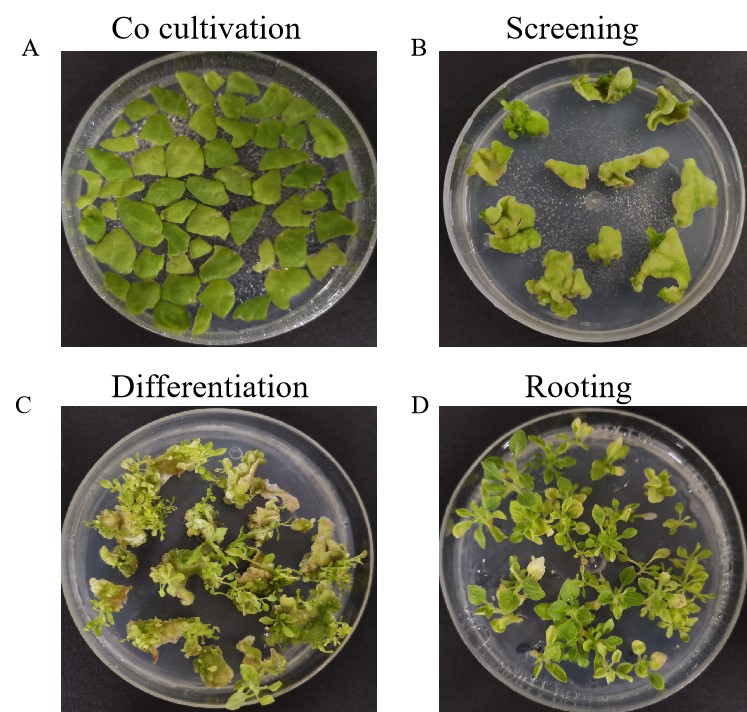


**Supplemental Fig. 3**  **The MnSOS3-pBWA(V)HS recombinant plasmid were transferred into Agrobacterium GV3101 by the tobacco leaf disc transformation method**

*
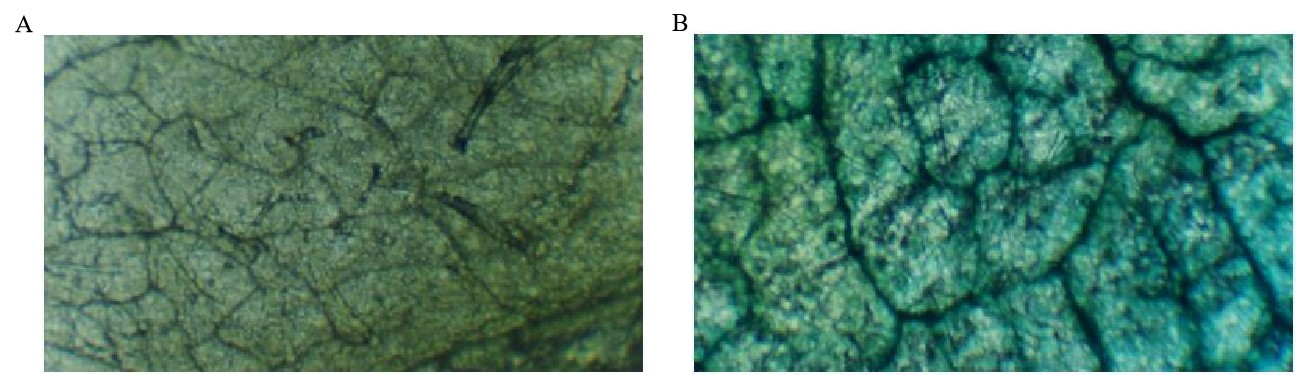
*

**Supplemental Fig. 4**  **GUS staining of transgenic tobacco leaves and control tobacco leaves cells.**

**（A）Control tobacco leaves （B）Transgenic tobacco leaves**
