# Supplementary material for: Analysis of salt resistance conferred by salt overly sensitive 3 protein from mulberry (Morus notabilis)
Source: Front Plant Sci. 2026 Jan 30;17:1694392. doi: 10.3389/fpls.2026.1694392 (PMC12900689; doi:10.3389/fpls.2026.1694392)
Supplement: Supplementary file 2 [file Table1.docx]

# Supplemental Table 1: Primers used in this study

| Gene | Primer (5'-3') | Used for |
| --- | --- | --- |
| *MnSOS3* | ATGGGCTGCTTTTCGTCCAAGCAAG | RT-PCR and transgenic screening |
|  | CACATCTGAATCTTCCATTTCCGAG |  |
| *MnSOS3* | AACACGGGGGACTTTGCAACatgggctgcttttcgtccaagcaag | Construction of pBWA(V)HS-MnSOS3 |
|  | TGAAGACAGAGCTAGTTACAttacacatctgaatcttccatttccgagttcattac |  |
| *MnSOS3* | TTCTTGCCGCTGAAACAC | q-PCR |
|  | GTGCCAACACCATCTCCTT |  |
| *MnSOS3* | CTGCCCGCTGTTCTACAACCGG | pBWA(V)HS-MnSOS3 detection primers |
|  | GGAGCATATACGCCCGGAGTC |  |
| *MnSOS3* | ctcggtaccctcgagATGGGCTGCTTTTCGTCCAAGCAAG | Prokaryotic expression |
|  | tctagactgcaggtcCACATCTGAATCTTCCATTTCCGAG |  |
| *MnSOS3* | AACACGGGGGACTTTGCAA | Sequencing primer |
|  | TGAAGACAGAGCTAGTTACA |  |
| *MnSOS2* | ATGAAGAAGGTAACAAGGAAGGTCG | RT-PCR |
|  | TCAGCAAGTCATGGTTCTAAGCAGG |  |

Note: Lowercase letters represent the homology arms containing *Bamh 1* or *sal 1* restriction sites
