# Supplementary material for: Analysis of salt resistance conferred by salt overly sensitive 3 protein from mulberry (Morus notabilis)
Source: Front Plant Sci. 2026 Jan 30;17:1694392. doi: 10.3389/fpls.2026.1694392 (PMC12900689; doi:10.3389/fpls.2026.1694392)
Supplement: Supplementary file 3 [file Table2.doc]

**Supplemental Table 2：Na⁺and K⁺contents in leaves and roots of transgenic (*MnSOS3*-2) and WT**

| Tissue | Treatment | Na⁺ (TP vs. WT ) | K⁺ (TP vs. WT ) | Na⁺/K⁺ Ratio (TP vs. WT ) |
| --- | --- | --- | --- | --- |
| Leaves | Control | 0.15 ± 0.08 vs. 0.14 ± 0.05 | 2.10 ± 0.02 vs. 2.08 ± 0.01 | 0.07 ± 0.00 vs. 0.07 ± 0.00 |
|  | 250 mM NaCl | 0.95 ± 0.02 vs. 2.10 ± 0.07** | 1.75 ± 0.02 vs. 1.20 ± 0.02** | 0.55 ± 0.01 vs. 1.71 ± 0.08** |
| Roots | Control | 0.25 ± 0.01 vs. 0.26 ± 0.01 | 1.80 ± 0.02 vs. 1.82 ± 0.01 | 0.14 ± 0.01 vs. 0.14 ± 0.00 |
|  | 250 mM NaCl | 4.81 ± 0.02 vs. 3.52± 0.03** | 1.55 ± 0.02 vs. 1.10 ± 0.02** | 3.10 ± 0.03 vs. 3.19 ± 0.03 |

Note: Sodium chloride treatment time is 24 h. Data are means ± SD (n=3). **p < 0.01 compared to control. units：mmol/g FW
