# Supplementary material for: Analysis of salt resistance conferred by salt overly sensitive 3 protein from mulberry (Morus notabilis)
Source: Front Plant Sci. 2026 Jan 30;17:1694392. doi: 10.3389/fpls.2026.1694392 (PMC12900689; doi:10.3389/fpls.2026.1694392)
Supplement: Supplementary file 4 [file Table3.doc]

**Table S3 | Media formulations for *E. coli* and yeast.**

*E. coli*Media:
LB: 0.5% (w/v) yeast extract, 1% (w/v) polypeptone, 1% (w/v) NaCl.
LB+Amp: LB medium supplemented with 100 μg/mL ampicillin.
LB+Kan: LB medium supplemented with 50 μg/mL kanamycin.

Yeast Media:
YPDA: 1% (w/v) yeast extract, 2% (w/v) tryptone, 2% (w/v) glucose, 0.02% (w/v) adenine.
Synthetic Drop-out (SD) Media: Prepared according to the Clontech Yeast Protocols Handbook (PT3024-1).
SD/-Trp: Synthetic Defined medium lacking Tryptophan.
SD/-Leu: Synthetic Defined medium lacking Leucine.
DDO: SD/-Leu/-Trp (Double Dropout).
TDO: SD/-Leu/-Trp/-His (Triple Dropout).
QDO: SD/-Leu/-Trp/-His/-Ade (Quadruple Dropout).
TDO/X: TDO medium supplemented with X-α-Gal.
QDO/X: QDO medium supplemented with X-α-Gal.
